# The Retribution-Gap and Responsibility-Loci Related to Robots and Automated Technologies: A Reply to Nyholm

**DOI:** 10.1007/s11948-019-00120-4

**Published:** 2019-07-02

**Authors:** Roos de Jong

**Affiliations:** Rathenau Instituut, The Hague, The Netherlands

**Keywords:** Agency, Responsibility-gaps, Retribution-gaps, Human–robot collaborations

## Abstract

Automated technologies and robots make decisions that cannot always be fully controlled or predicted. In addition to that, they cannot respond to punishment and blame in the ways humans do. Therefore, when automated cars harm or kill people, for example, this gives rise to concerns about responsibility-gaps and retribution-gaps. According to Sven Nyholm, however, automated cars do not pose a challenge on human responsibility, as long as humans can control them (even if only indirectly) and update them. He argues that the agency exercised in automated cars should be understood in terms of human–robot collaborations. This brief note focuses on the problem that arises when there are multiple people involved, but there is no obvious shared collaboration among them. Building on John Danaher’s discussion of command responsibility, it is argued that, although Nyholm might be right that autonomous cars cannot be regarded as acting on their own, independently of any human beings, worries about responsibility-gaps and retribution-gaps are still justified, because it often remains unclear how to allocate or distribute responsibility satisfactorily among the key humans involved after they have been successfully identified.

## Introduction

The first serious crashes with automated cars on public roads occurred in 2016. In February a Google self-driving car collided with a public bus. In May a fatal accident took place as a Tesla Model S on “autopilot” hit the side of a tractor trailer. Whereas Google accepted partial responsibility for what happened (Urmson 2016), Tesla at the time emphasised that Model S customers would need to assume “control and responsibility” (Tesla 2016). Nevertheless, both Google and Tesla promised to update the software of their cars, so as to make them better able to handle comparably dangerous situations. More recently, in early 2018, Uber announced that it is temporally suspending its programme with self-driving cars after one of its vehicles was involved in a tragic incident in Arizona, marking the first pedestrian fatality (abc15 2018). When people are harmed or killed by automated technologies in similar ways, who ought to be held responsible?

In a recent article, Nyholm (2017) refers to the first two above-mentioned cases as he examined how to allocate responsibility when automated technologies or robots harm or kill people. Other authors have also raised concerns about responsibility-gaps because automated systems are making decisions that cannot be fully controlled or predicted (Coeckelbergh 2016; Gunkel 2017; Matthias 2004; Sparrow 2007). Traditional concepts of responsibility ascription appear to be inadequate in these new situations. Moreover, automated systems cannot respond to punishment and blame in the ways humans do. Their level of independence and autonomous power may therefore ultimately give rise to what Danaher (2016) calls retribution-gaps: a desire for retribution without appropriate subjects of retributive blame. However, according to Nyholm, automated cars *do not* pose a challenge to human responsibility, as long as people can control them (even if only indirectly) and update them (Nyholm 2017). To make his case, Nyholm critically analyses the types of agency that can and cannot be attributed to robotic systems. He argues that the agency exercised in automated cars should be understood in terms of *human*–*robot collaborations*.^1^ The main question to be asking is which of the humans involved are *most* responsible.

This article focuses on the problem that arises when there are multiple people involved, but there is no obvious *shared* collaboration among the different individuals involved.^2^ Although I agree with Nyholm that autonomous cars cannot be regarded as acting on their own, independently of any human beings, I contend that worries about responsibility-gaps and retribution-gaps are still justified. In real-world (non-ideal) situations it is likely unfeasible to adequately trace harmful outcomes to one or more particular persons involved in circumstances leading to the accident. Moreover, solely focusing on the role of people does not do justice to the “complex” constituted by humans and things (Latour 1992; Verbeek 2011). It often remains unclear how to allocate or distribute responsibility satisfactorily among the key humans involved after they have been successfully identified.

## Who is to Blame?

Before looking into the human responsibilities, it is helpful to examine carefully the issues behind whether automated cars (or robots) themselves are responsible for morally harmful outcomes. Although it has been theorised that certain technologies could qualify as “actors” (Akrich 1992; Latour 1992) or even as moral agents (Floridi and Sanders 2004), Nyholm argues that relevant differences in types of agency need to be distinguished from each other. When humans use machines—such as automated cars—a hierarchical collaborative agency is involved. Even though the car might be doing “most of the work,” the goals are set by another authoritative agent; the humans involved initiate, supervise, and manage the agency of their robotic collaborators.^3^ Therefore, it is the human collaborator who should be held responsible (Nyholm 2017).

John Danaher (2016) seems to take a different route, focusing instead on the attitudes and responses typically associated with moral responsibility. According to him, robots will not be appropriate targets of retributive blame because, even though they could be causally responsible for an injurious outcome, they do not have the mental capacities (i.e. beliefs, desires, intentions) or the moral faculties or sensitivity to moral reasons for action that open them up to blame (Danaher 2016). Danaher also notes that people are generally unsatisfied with ascribing blame to non-human-like agents.

The different lines of reasoning show that, whereas Danaher is looking for a culpable wrongdoer deserving of punishment, Nyholm does not elaborate on the *psychological desire* to punish. Instead, Nyholm focuses on the allocation of responsibility with regard to attributions of different types of agency.

Interestingly, the different approaches of the two authors lead to diverging conclusions with regard to the responsibility of manufacturers, designers or other associated human agents. Similar to Andreas Matthias (2004), Danaher argues that the degree of autonomy in automated cars opens up liability- and retribution-gaps (Danaher 2016). According to Danaher, it is evident that although the manufacturer or designer has a duty of care, the involvement of machine learning algorithms poses difficulties as to how to interpret the standard of care. Even though vicarious liability rules and strict liability rules could ensure that somebody is *held responsible* and people receive the necessary compensation, it might not *feel right* to blame the programmer who could not anticipate, expect, or reasonably foresee the actions of the car. Resolving the issue “who will pay for the wrongdoing of the robot?” is easier than determining “who deserves retributive blame?”.

It is important to keep in mind that Danaher, in contrast to Matthias, does not assume great degrees of autonomy in automated cars per se.^4^ As he sees things, the problem already arises as soon as the car is able to break, turn, and accelerate across a range of environments, without the *need* for human interference or control. For Matthias the problem lies with artificial learning systems which act by rules that are not fixed *by people* during the production, but can be changed *by the machine* during the operation (Matthias 2004). Nyholm, however, is convinced that as long as people *can* interfere, for example in terms of stopping or updating the car, there will not be such a responsibility-gap.

Nyholm suggests focusing on what he calls the key responsibility-loci. He argues there is a set of questions that can help in this regard. One should ask: (1) under whose supervision or control the vehicle is operating, (2) who is currently able to start, take over, or stop the car, (3) whose preferences regarding driving-style the car is conforming to, (4) who is better situated to observe and monitor the car’s behaviour on the road, and (5) who understands the functioning of the car. When the answers to these questions are decided, it will also be possible to determine which humans are *most* responsible for the actions the car performs (Nyholm 2017).^5^

However, suppose there is one person capable of stopping the car, another who can update the car’s computers, and yet another who is best situated to observe and monitor the car. One can even suppose that there is yet another person to whose preference the car’s functioning is made to conform. In such a case it might become challenging to attribute responsibility. What appears to be problematic here is that the answers to the set of questions proposed by Nyholm can point in different directions.

Nyholm briefly discusses an interesting scenario of this sort himself (Nyholm 2017): an automated car could be executing the human driver’s particular travelling goals (e.g., going to the grocery store), while the car-company determines the means by which that end is achieved (e.g., determining the route). In this case two sets of human–robot collaborations are involved, rather than an obvious form of shared collaboration. The “driver-car” collaboration and the “programmer-car” collaboration have their own goals and are not quite on the same team or part of one line of command. Whatever is in their respective best interests may also differ. This makes it challenging to determine which of the humans involved is most responsible for the actions the car performs.

When the automated car crashes halfway on the route to the grocery store—just by being at the wrong place at the wrong time—should the human commander who set this goal be blamed or the human behind the navigating software? Even though Nyholm acknowledges that such a scenario gives rise to difficult questions, he does not seem willing to admit that this might ultimately give rise to responsibility-gaps or retribution-gaps. What this all shows is that these gaps cannot always simply be filled by arguing that machine agency should be understood as a kind of collaborative agency in which automated machines are typically best understood as participating in human–robot collaborations. Nor is it enough to have answers to Nyholm’s set of questions. The reason, to repeat, is this: one and the same robotic agent may sometimes simultaneously participate in more than one human–robot collaboration, and the circumstances may be such that the key humans involved cannot plausibly be seen as collaborating with each other in a way that makes them jointly responsible for the outcomes of what the robot does.^6^ Ironically, it is precisely by reflecting on different possibilities regarding how the set of questions that Nyholm himself proposes might be answered that one can most easily come to see this.

## Danaher on Command Responsibility

To further clarify the point made above and what is at stake, it will be helpful to turn to Danaher’s discussion of the Command Responsibility Objection (Danaher 2016). Nyholm seems to argue for adopting stricter liability standards or a new regime of responsibility norms in which human commanders take responsibility for any misdeeds of their robotic collaborators (compare: Joanna Bryson (2010) argues that robots should be built, marketed and considered legally as slaves). A clear public announcement of such norms may plug the retribution-gap (Danaher 2016). However, Danaher convincingly indicates that there are several potential pitfalls in this approach: (1) the strict standard of command responsibility might fail to align with what is judged to be retributively appropriate,^7^ (2) imposing too high a standard of responsibility might have a stultifying effect on the (potential socially beneficial) development of robots, and (3) it is difficult to impose a command responsibility framework onto fragmented and distributed organisations. Even though the second point about slowing down or even completely blocking the development of automated cars is interesting and has been taken up by other authors as well (e.g., Gunkel 2017),^8^ the present discussion focuses on the other two as they have a clear impact on how potential responsibility can be allocated.

The first pitfall is most likely to appear if it is decided upfront that, for instance, the companies that manufacture and produce the cars (e.g., the senior management of Google or Tesla) always have command responsibility. In case of a crash, it could reasonably be the case that there were other factors involved that really made any form of anticipating what happened close to impossible. Generally, people might therefore deem it unfair or disproportionate to ascribe a level of retributive blame to one particular “commander” that covers the full gravity of the moral harm done. However, by adopting this new standard of blame attribution, in a legal framework for instance, everyone knows beforehand that the companies would have command responsibility no matter what (Danaher 2016).

If it turns out that the strict standard of responsibility really does not comply with widely shared intuitions of retributive justice, one could either accept this discrepancy or apply a more relaxed doctrine. If one sticks to the strict standard, it can lead to controversy as it means that the legal punishment does not fit with what is retributively appropriate in the eyes of many people. If one allows deviation from the implications of strict command responsibility, however, there can be problems also, as the degree of blame is then likely to be seriously attenuated. As Danaher points out, there is no gap in the human willingness to assign blame. Consequently, “there is a level of harm that is unmatched by a proportionate or corresponding level of retributive blame” (Danaher 2016, 305).

At first glance, Nyholm’s approach seems to be able to tackle this challenge without stretching the existing standards of blame attribution. After all, Nyholm does not seem to suggest that only *one* person needs to be blamed. If one assumes that, following Nyholm, it is taken as a starting point that the mere presence of unpredictability and a lack of direct control are not by themselves enough to create responsibility-gaps, then it is only necessary to ensure that all key humans involved are accurately identified. Once all human players are identified, the appropriate level of blame can be ascribed to each and every one of them. As long as the distributed blame still adds up to the appropriate level of reattributed blame, the attenuation problem can potentially be avoided. Rather than simply giving the senior management of the company what might be called blanket command responsibility, the command responsibility framework proposed by Nyholm seemingly covers all key humans involved. Moreover, Nyholm seems to assume that any possible defect, accident or case of bad luck can ultimately be traced back to a particular person. For the sake of argument, it is a reasonable assumption.

## Fragmented and Distributed Responsibility

It may be that, in theory and in a highly idealised set of conditions, what Nyholm proposes is indeed the correct way to approach the problem. Identifying responsibility-loci among all humans involved may succeed to align with what people believe is, morally speaking, right. However, this will be a difficult thing to do in practice. The so-called “problem of many hands” (van de Poel and Fahlquist 2012) is another important stumbling block for the approach Nyholm proposes. This links nicely with another potential pitfall for attempts to fill retribution-gaps with the help of the notion of command responsibility described by Danaher (2016). Automated cars—their physical components, algorithms, software, etc.—are often developed by complex, distributed networks, with no clear hierarchy or visible infrastructure.^9^ This will make it difficult to determine who has command responsibility in advance.

The crucial practical question here is this: when the key humans involved are not part of one well-integrated, large organisation, how should one then distribute responsibility? Whenever there is any sort of fragmented and distributed responsibility, it is less than obvious how to design management structures for vicarious liability. I concur that it is important to identify the key humans involved, but suggest that the potential pitfalls as described by Danaher need to be taken seriously as well. Especially in the scenario described earlier in which one person is setting the more particular goal and another person or set of persons is responsible for the means to achieve the goal, there is no obvious form of shared collaboration. If, on top of this, (part of) the software, for instance, is developed by several groups who are also not necessarily “on the same team” either, it will be even more challenging to avoid responsibility-gaps and retribution-gaps.

## Conclusion

To sum up, when there is no obvious shared collaboration but instead several humans are involved in different human–robot collaborations, this typically makes it challenging to identify satisfactorily a single player who has command responsibility. Identifying which humans are most responsible, and giving all of them a certain portion of command responsibility, does not seem to solve all issues either. Furthermore, this approach would defeat the purpose of implementing strict liability rules. Since none of the humans involved have individual control over the car and its behaviours, different levels of blame will have to be distributed across a broad range of individuals.

The first challenge this gives rise to is to make sure that blaming everyone a little ultimately adds up to the appropriate total amount of retributive blame. The second challenge is to successfully trace back *who* is actually responsible for *what* when many people are involved that are not part of one shared organisation with a clear hierarchy. Merely installing a legal framework for dealing with responsibility surely does not resolve the psychological and moral issue about just deserts. A gap might still arise between the general desire to find appropriate targets of retributive blame and what people believe to be, morally speaking, right (e.g., what is retributively appropriate). Identifying multiple responsibility-loci does not ensure that appropriate targets of blame will be found. It is therefore fair to conclude that even when the key human players involved have been identified, responsibility-gaps and retribution-gaps are not yet plugged.

## Footnotes

1 It is noteworthy that Nyholm’s discussion of *human*–*robot collaborations* is different from Peter-Paul Verbeek’s post-phenomenological work on *human-technology associations* (Verbeek 2011). Verbeek argues that moral agency should be understood as a fundamentally hybrid affair. Moral agency, according to Verbeek, cannot be located exclusively in technology *itself*, nor exclusively in humans (Verbeek 2014, 80). By arguing that moral agency is distributed among humans and nonhumans, he is not saying that a form of agency (or blame for that matter) should be attributed to technologies that would normally only be attributed to human beings. Instead he claims “seeing the moral significance of technologies makes us more responsible, rather than less” (Verbeek 2014, 85). Similar to Verbeek, Nyholm argues that certain types of agency cannot be attributed to technologies and that humans are ultimately responsible. However, Nyholm does not seem to fully embrace Verbeek’s theory of mediated agency as throughout his paper he seems to assume (and to be in favour of strictly applying and enforcing) an instrumental theory of technology (i.e. technologies serve the purposes of users; its agency is based on human goals or wishes). Nyholm discusses interactions between humans and technologies as a type of collaborative agency of a hierarchical sort, where certain responsible humans occupy the roles of supervisors or commanders. He does not (explicitly) reflect on how the technologies might help shape what people do and want, nor how they could change what people can be responsible for.

2 The point here is somewhat related to the so-called problem of many hands which occurs when many people are involved in an activity and the joint effect is an undesired state-of-affairs which was not directly caused or intended by any of them (van de Poel and Fahlquist 2012). However, when a particular robot participates in different human–robot collaborations at one and the same time, it becomes even more complicated to properly allocate responsibility.

3 It seems that Nyholm’s conception of “supervision” differs from that of Matthias’ (2004). Matthias claims there is an increasing number of situations in which technologies act without human supervision: it can be either in principle or, for economic reasons, impossible for a human expert to supervise an operating machine (e.g., because of an information advantage of the machine, its processing speed, and the multitude of operational variables involved) (Matthias 2004). Nyholm focuses on the sorts of functions the systems can perform. Ultimately, the machine is under the watch of a human who could step in and stop the machine. Presumably, Matthias would not disagree with this observation, but he argues that humans face a responsibility-gap exactly because stopping (or not using) self-learning machines is sometimes not a realistic option.

4 It is interesting to note that Danaher’s article “The Rise of the Robots and the Crisis of Moral Patiency” elaborates on the worry that outsourcing more and more tasks to robots and artificial intelligence might also result in outsourcing responsibility. Danaher argues that even though technology will not rob humans of their status as moral agents, they should reflect on what tasks they are willing to outsource (Danaher 2019).

5 These questions seem very much focused on checking whether the technology is doing only what it is programmed to perform. Gunkel (2017) elaborates on recent innovations with regard to autonomous technology, machine learning and social robots that challenge the instrumental theory of technology and open up responsibility-gaps. Gunkel explains that assigning some level of moral agency to machines, distributing responsibility across humans and nonhumans (as proposed by Verbeek), and going for a strict application of the instrumental theory are three options that could help remediate the gap. However, they all have their advantages and disadvantages.

6 The problem of many hands clarifies the difference between individual and collective agency and responsibility. It can be difficult both to discern individual contributions (how each actor contributed to the undesired state-of-affairs) in the first place, and what the joint causal responsibility implies with regard to moral responsibility of the individuals (van de Poel and Fahlquist 2012, 898). Notably, in some cases it seems that the collective can be held responsible, but not any of the people involved individually (Pettit 2007).

7 For the purpose of this paper it is not necessary to take a stance with regard to what is or is not retributively appropriate. However, it is important to distinguish between acknowledging the emotional retributivist disposition of (not necessarily all) people on the one hand, and making claims about whether the desire for retribution is *justified*, on the other. In other words, working within Danaher’s framework, there is the possibility that people might *feel* there is an injustice if harm or injury are dealt with in a way that does not fit with people’s intuitions.

8 Interestingly, Gunkel predicts developers might want to protect themselves from culpability, whereas it appears that several car companies actually have adopted the business strategy to explicitly take responsibility for the actions of the car. Volvo, Google and Mercedes have already announced that they will accept full responsibility if one of their autonomous cars causes a collision (Elmer 2015).

9 This is already the case with regular cars of course. Software also plays a role in normal cars and the manufacturing of different parts is often spread out among companies all over the world. However, automated cars might involve even more distributed responsibility. Currently there stills seems to be a clear hierarchy; whoever designs and makes the outside of the car (and the engine), or whoever puts together all parts in the end is considered the key player. The autonomous cars might change the chain of command between software engineer and designers.
